# Drug-drug interaction identification using large language models

**DOI:** 10.64898/2025.12.03.25341549

**Published:** 2025-12-29

**Authors:** Kaitlin Blotske, Xingmeng Zhao, Kelli Henry, Yanjun Gao, Adeleine Tilley, Moriah Cargile, Brian Murray, Susan E. Smith, Erin F. Barreto, Seth Bauer, Sunghwan Sohn, Tianming Liu, Tell Bennett, Mitch Cohen, Andrea Sikora

**Affiliations:** University of Colorado School of Medicine, Department of Biomedical Informatics; University of Colorado School of Medicine, Department of Biomedical Informatics; University of Colorado School of Medicine, Department of Biomedical Informatics; University of Colorado School of Medicine, Department of Biomedical Informatics; University of Colorado Anschutz Medical Campus, Skaggs School of Pharmacy and Pharmaceutical Sciences. Department of Clinical Pharmacy, Aurora, CO, USA; University of Colorado Anschutz Medical Campus, Skaggs School of Pharmacy and Pharmaceutical Sciences. Department of Clinical Pharmacy, Aurora, CO, USA; University of Colorado Anschutz Medical Campus, Skaggs School of Pharmacy and Pharmaceutical Sciences. Department of Clinical Pharmacy, Aurora, CO, USA; University of Georgia College of Pharmacy, Department of Clinical and Administrative Pharmacy, Athens, GA, USA; Mayo Clinic, Rochester, MN, USA; Cleveland Clinic, Cleveland, OH, USA; Mayo Clinic, Rochester, MN, USA; Department of Computer Science, University of Georgia, Athens, GA; University of Colorado School of Medicine, Department of Biomedical Informatics Aurora, CO, USA; University of Colorado Anschutz Medical Campus, Department of Surgery; University of Colorado School of Medicine, Department of Biomedical Informatics; University of Georgia College of Pharmacy, Department of Clinical and Administrative Pharmacy, Augusta, GA, USA

**Keywords:** large language model, artificial intelligence, healthcare, medications, pharmacy

## Abstract

**Background:**

Drug-drug interactions (DDIs) are a significant source of morbidity and adverse drug events (ADEs), particularly in situations of polypharmacy and complex medication regimens. While rules-based software integrated in electronic health records (EHRs) has demonstrated proficiency in identifying DDIs present in medication regimens, large language model (LLM) based identification requires thorough benchmarking and performance evaluation using high-quality datasets for safe use. The purpose of this study was to develop a series of performance benchmarking experiments specifically for LLM performance in identification and management of DDIs using a specifically curated clinician-annotated dataset of clinically-relevant DDIs.

**Methods:**

We evaluated three LLMs (GPT-4o-mini, MedGemma-27B, LLaMA3–70B) using a clinician-annotated benchmark dataset of 750 DDI scenarios spanning three levels of diagnostic complexity. Tasks were aligned with flexible judgment formats: (1) a pointwise two-drug classification task, (2) a pairwise three-drug discrimination task, and (3) a listwise 4–6 drug selection task. Standardized zero-shot prompting with task-specific instructions was applied for all models. Performance was assessed using precision, recall, F1 score, and accuracy. Reliability was quantified using self-consistency across repeated runs and confidence-aligned metrics to capture stability in model reasoning.

**Results:**

Across the three experiments, model performance varied by task structure and interaction severity. LLaMA3–70B demonstrated the highest recall and F1 score in the pointwise task, whereas GPT-4o-mini achieved superior accuracy and consistency in the pairwise and listwise tasks. MedGemma-27B showed competitive performance in identifying Category D interactions. Self-consistency decreased as task complexity increased, highlighting reduced stability in multi-drug reasoning. No model exhibited uniformly high reliability across all judgment formats.

**Conclusions:**

Current LLMs show promising but uneven capabilities in identifying DDIs across clinically relevant task structures. Performance degrades as the reasoning space expands, and stability across repeated queries remains limited. These findings emphasize the need for multi-format evaluation frameworks and reliability-aware assessment when considering LLMs for medication-safety applications.

## Background

Clinical natural language processing (cNLP), particularly in the form of large language models (LLMs), has completed impressive feats in the healthcare space, and great interest exists for how these new technologies can improve healthcare, including medication safety. Importantly though, a recent Federal Food and Drug Administration (FDA) statement was published regarding artificial intelligence (AI) regulation stating: “The sheer volume of these changes and their impact also suggests the need for industry and other external stakeholders to ramp up assessment and quality management of AI across the larger ecosystem beyond the remit of the FDA…all involved sectors will need to attend to AI with the care and rigor this potentially transformative technology merits.”^1^

Careful probing and documentation of LLM performance is imperative for safe and efficacious deployment for medication-related tasks. Previous studies have evaluated LLMs in the space of drug-drug interaction (DDI) support further investigation and development of this technology but also identified errors that have substantial potential to impact patient safety.^2–17^ However, none of these studies have been targeted at benchmarking performance with the goal that LLMs could be said to have safe and effective performance for clinical use. Importantly, the generated datasets that have been used prior have been reviewed and demonstrated inaccuracies or lack of clinical relevance.^18,19^ Notably, DDI-Corpus, one of the largest DDI databases with over 5,000 DDIs, was developed using DrugBank and Medline abstracts but had no clinical validation as to the relevance of these DDIs, resulting in a large portion of the listed DDIs containing conflicting information (for example, nonsteroidal anti-inflammatory drugs [NSAIDs] and aspirin listed as “no relation” and “advise” depending on the referenced article) or incorrect information (for example, “warfarin” and “coumadin” listed as having no relation).^18^ Additionally, many of the medications included are outdated and not available on the market.^18^ Several LLMs have used DDI-Corpus for training and evaluation of DDIs, but caution is warranted for the application of their results given the lack of clinical validity in the training dataset.^20^ It is also imperative to note that standard, rules-based computer applications and software (e.g., Lexi-Drugs) has perfect performance in the identification of DDIs housed within their database when queried.

The purpose of this study was to develop a series of performance benchmarking experiments specifically for LLM performance in identification and management of DDIs using a curated clinician-annotated dataset of clinically-relevant DDIs.

## Methods

### Study design.

We designed a three-part evaluation framework to assess how LLMs identify DDIs across tasks that mirror real clinical decision-making. Three models were evaluated: GPT-4o-mini, MedGemma-27B, and LLaMA3–70B. To capture different aspects of DDI reasoning, we implemented three complementary judgment formats, pointwise, pairwise, and listwise. These formats have been widely adopted in recent LLM-as-judge literature. These formats allow assessment of single-item correctness, comparative discrimination among alternatives, and multi-option selection, providing a more complete view of model behavior. This structure follows the logic of “multi-format judgment” introduced in tool-integrated LLM judge frameworks and has been shown to reveal different failure patterns that would be missed using only a single evaluation mode. Each experiment was run in triplicate. This project was reviewed and approved by the University of Colorado Institutional Review Board (COMIRB #25–1631). All methods were performed in accordance with the ethical standards of the Helsinki Declaration of 1975.^21^ This evaluation followed the transparent reporting of a multivariable model for individual prognosis or diagnosis (TRIPOD–LLM) extension reporting frameworks, as applicable (Supplemental Appendices 1).^22^

### Dataset Development.

A clinician-curated dataset of 750 unique DDI scenarios was developed. Interaction labels were sourced from LexiDrug, which provides standardized definitions for Categories A (no known interaction), B (no action needed), C (monitor therapy), D (consider therapy modification), and X (avoid combination).^23^ A total of 250 scenarios were developed for each of the following 3 experiments. The two-drug experiment consisted of interacting (C/D/X) and non-interacting (A) drug pairs. The three-drug experiment included one target drug and two candidate drugs, exactly one of which produced a known interaction. The 4–6 drug experiment contained short medication lists with a single interacting drug pair concealed within each list. Dataset construction followed the clinical-validation process used in prior pharmacology-oriented LLM research: all cases were independently reviewed by three board-certified clinical pharmacists, and disagreements were resolved by consensus.{Albogami, 2024 #35;Chase, 2025 #62;Hsu, 2023 #22;Huang, 2024 #33;Munir, 2024 #36;Thapa, 2025 #41;van Nuland, 2024 #32;Zhou, 2025 #50} This ensured that the benchmark reflected *clinically relevant* DDIs with accurate severity classifications. These datasets are posted on Github: https://github.com/sikora07/AIChemist. Characteristics of each dataset are summarized in Table 1.

### Judgment Formats and Prompting Procedure.

All models were evaluated using standardized zero-shot prompts tailored to the specific structure of each task (Table 2). To minimize parsing errors and hallucinations, prompts incorporated rigorous clinical definitions derived from standard interaction categories (e.g., ‘Avoid combination’, ‘Monitor therapy’) and enforced strict output formats, specifically, a binary ‘A’/’B’ selection or a structured JSON object. To ensure the reliability of our results and account for the stochastic nature of Large Language Models, we employed a robust repeated-measures design. Each unique prompt was queried across nine independent runs under identical sampling parameters (temperature=0.7). This extensive repetition allowed us to distinguish between stable clinical reasoning and stochastic variation, filtering out “lucky guesses” from robust knowledge. To further mitigate artifacts such as position bias (the tendency of LLMs to prefer the first option presented), we implemented a label shuffling mechanism. Across the nine runs, the assignment of semantic labels (e.g., CORRECT/INCORRECT for pointwise tasks or Candidate A/Candidate B for discrimination tasks) to the output tokens (‘A’ or ‘B’) was randomized. This ensures that reported accuracy reflects true semantic understanding rather than token preference.

For the pointwise evaluation task, we adopted a balanced negative sampling strategy in which a negative example represents a counterfactual clinical claim, meaning a valid drug pair is paired with an incorrect drug–drug interaction (DDI) category (for example, assigning a Category X label to a pair whose verified category is Category C). Clinically, such counterfactuals reflect misleading or unsafe interaction assessments that may cause inappropriate avoidance of safe combinations or failures to identify high-risk contraindications. For each ground-truth positive instance, we procedurally generated a matched negative instance by randomly selecting an incorrect category for the same pair, resulting in a one to one positive to negative distribution. This design requires the model not only to recognize correct interaction labels but also to detect and reject clinically implausible or unsafe categorizations, which tests its ability to identify false clinical assertions, recognize contradictions with established pharmacological evidence, and minimize the effects of acquiescence bias.

#### Performance Metrics.

Performance metrics were selected to align with the objectives of each experiment. All metrics were computed at the **clinical case level**, where a *case* refers to the full input provided to the model (e.g., a single drug pair, a three-drug prompt, or a 4–6 drug list). Each case yielded exactly one prediction and therefore contributed a single accuracy or error outcome, regardless of how many model tokens or intermediate steps were generated.

For the two-drug classification task (pointwise setting), we report accuracy, precision, recall, and F1.

- Accuracy measured the proportion of correctly classified cases and was computed as:
$$\frac{\left(TP\;+\;TN\right)}{\left(TP\;+\;TN\;+\;FP\;+\;FN\right)}$$
- Precision measured the proportion of positive predictions that were correct:
$$\frac{TP}{\left(TP\;+\;FP\right)}$$
- Recall measured how many true interactions were successfully identified:
$$\frac{TP}{\left(TP\;+\;FN\right)}$$
- The F1 score, which balances precision and recall, was calculated as
$$2\times \frac{\left(\mathrm{precision}\;\times \;\mathrm{recall}\right)}{\left(\mathrm{precision}\;\;+\;\;\mathrm{recall}\right)}$$

For the three-drug (pairwise) and multi-drug (listwise) tasks, accuracy was defined as selecting the correct interacting drug or the correct interacting drug pair for each complete case. Across all experiments, we also measured self-consistency to assess how reliably each model produced correct answers across repeated runs. Self-consistency was defined as number of runs that produced the correct answer divided by the total number of runs for that case. This metric captures whether a model consistently arrives at the correct answer when asked multiple times. A model that only occasionally produces the correct response will have low self-consistency even if its single-run accuracy appears acceptable, making this measure important for evaluating reliability in medication-safety tasks.

#### Statistical Analysis.

We evaluated model performance using each full clinical case as the unit of analysis. To quantify uncertainty in the reported metrics, we computed 95% confidence intervals using the percentile bootstrap with 1,000 resamples. In each bootstrap iteration, we drew a new sample of complete clinical cases with replacement and recalculated the metric of interest. We did not resample individual model outputs from the same case. This approach ensures that the confidence intervals reflect how model performance might change when applied to new drug–drug interaction cases, rather than capturing randomness from repeated generations of the same prompt. Given the hypothesis generating nature of this exploration, no attempt was made to calculate sample size. All analyses were performed using Hugging Face package.^24^

## Results

We evaluated three large language models, GPT-4o-mini, MedGemma-27B, and LLaMA3–70B across three DDI tasks. Performance for each task is summarized in Table 3. Overall, model behavior differed substantially across pointwise, pairwise, and listwise settings, with no model demonstrating uniformly superior performance. Patterns across experiments highlight both strengths and persistent limitations in clinical reasoning and stability.

### Drug Pair Experiment (Pointwise).

Performance varied widely across models when classifying interaction categories for isolated drug pairs. LLaMA3–70B demonstrated the strongest recall (61.7, 95% CI 51.7–71.6) and F1 score (59.7, 95% CI 56.0–63.4), indicating a greater ability to correctly identify interacting pairs. GPT-4o-mini showed the highest precision (65.7, 95% CI 62.8–68.8), suggesting fewer false-positive assignments, but had lower recall and F1. MedGemma-27B had the poorest performance across all metrics (Table 3), particularly in recall (38.2, 95% CI 29.9–46.6). Self-consistency was low for all models, with values ranging from 31.0 to 43.0, consistent with earlier observations that LLM outputs often shift across repeated queries even when the underlying case remains unchanged. These findings echo prior studies showing instability in LLM clinical judgments, especially when tasks require precise multi-class classification.

### Three Drug Combination Experiment (Pairwise).

Accuracy improved considerably when models were asked to choose between two candidate drugs. GPT-4o-mini achieved the highest overall accuracy (86.6, 95% CI 82.8–91.2), closely followed by MedGemma-27B (84.9, 95% CI 78.4–87.6) and LLaMA3–70B (81.5, 95% CI 93.2–98.4). Performance patterns were consistent across interaction categories, with models performing best on Category D and Category X interactions. GPT-4o-mini demonstrated particularly strong discrimination for Category D (92.3, 95% CI 85.9–97.4), while MedGemma-27B showed comparable accuracy for Categories C and X. Self-consistency values were higher than in the pointwise task (73.7–94.4). This finding aligns with prior LLM evaluation frameworks where reduced answer space improves stability.

### Four-to-Six Drug Combination Experiment (Listwise).

Performance decreased in the more complex listwise setting, in which models were required to identify the interacting pair from a broader medication list. Accuracy ranged from 71.3 (95% CI 66.4–77.6) for GPT-4o-mini to 80.0 (95% CI 75.2–85.2) for MedGemma-27B and 68.6 (95% CI 63.2–74.4) for LLaMA3–70B. All models showed their lowest accuracy in Category C interactions (58.3–73.8 across models), reflecting the difficulty of detecting moderate-risk interactions when combined with additional distractor drugs. Category X interactions generally showed higher accuracy across models (74.3–80.5), although still lower than observed in the pairwise task.

**Figure 1.**
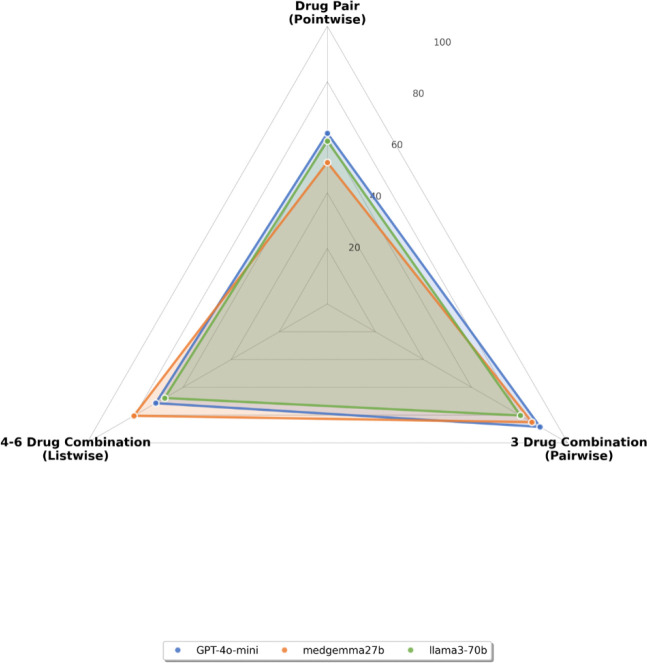
Radar Plot of Large Language Model Performance on Each Task Across all three models, the pairwise task is consistently easier than the pointwise task. This echoes patterns reported in other LLM evaluation work, where models tend to perform better when they can compare options directly rather than classify a single item in isolation. The results here follow the same trend: giving models two choices seems to reduce uncertainty and improve decision quality. Overall, the figure highlights that each model has a different “strength profile.”

As shown in Figure 1, the three models behave quite differently across the three DDI tasks. GPT-4o-mini shows the most even performance, staying relatively stable across the pointwise, pairwise, and listwise settings. MedGemma-27B performs best in the listwise task, where the model must identify an interacting pair from a larger set of drugs. This suggests that MedGemma-27B handles multi-drug reasoning more effectively than the other models. In contrast, LLaMA3–70B performs well in the pairwise task but drops noticeably in the listwise setting, indicating that its performance degrades as the number of candidate drugs increases.

## Discussion

This analysis marks the first time that a robust DDI dataset has been developed with clinical validation of all drug-drug interactions by clinicians in addition to validation by a reputable drug information source (LexiDrug).^23^ Previously published research on DDIs has focused on extraction methods to develop a database purely through extraction of DDIs from medical texts (ex. DDI-Corpus) and lacks the high-quality nature of a clinician-annotated dataset.^18,19,25–30^ Annotated DDI datasets are much rarer and previously have included annotations from experts with an informatics or biology background but have not included clinical annotations by experts in clinical medication use.^31^ A previous analysis did include an annotated dataset from a clinical pharmacist who selected relevant DDIs; however, this dataset only included DDIs for 5 medications (all macrolides or sodium-glucose transport 2 [SGLT2] inhibitors).^2^ Additionally, this study represents the first time that DDIs have been tested using a stepwise increase in complexity. Previous reports have included drug pairs with a simple “yes/no” response to “do these drugs have an interaction”.^2,7,8,20^. These studies did not include additional clinical context and reported highly variable performance. Our study specifically included severity of drug/drug interaction for the drug-pair experiment and added increased complexity by providing a list of possible drugs. Several previous studies have included lists of medications (multiple medications per patient case) but did not consistently report the number of medications or compare LLM efficacy in identifying DDIs when the number of medications to select from increased.^4,12,14,16,32^

Self-consistency scores declined across all models, emphasizing that increasing task complexity leads to greater variability in model reasoning and lower reliability. This pattern mirrors observations in prior research [1], where reasoning stability deteriorates as the number of candidate options increases. Across the three experiments, several consistent patterns emerged. First, models performed better in structured decision settings with limited answer space, as seen in the pairwise task. Second, performance was consistently lower for moderate-risk Category C interactions, reflecting the subtlety and context dependence of these cases. Third, self-consistency remained imperfect even in tasks with high accuracy, indicating that correct answers do not guarantee stable reasoning. This finding aligns with prior work demonstrating that LLMs often produce confident but shifting outputs, and that performance metrics alone can mask underlying instability.^33^

Several studies have evaluated LLM performance with real-world patient cases and/or medication profiles; one study demonstrated a low true positive rate and noted low agreement between GPT-3.5 and practicing pharmacists,^12^ while other studies have shown impressive DDI identification (ranging from 75.6–100%), but poor identification of the severity of the interaction.^14–16^ Our study mirrored this finding, as we noted worse performance on the drug pair (pointwise) task compared to previous studies, likely due to our prompt specifying the category of the DDI severity, as opposed to simply if a DDI exists between the two medications.^20^ Few studies have evaluated the proficiency of LLMs in making clinical recommendations: a study found that ChatGPT in response to sample textbook cases achieved only 70% agreement with clinical pharmacists,^13^ and in two studies of real patient cases, ChatGPT provided appropriate recommendations for management of DDIs 88% and 61.5% of the time.^4,32^ Overall, LLMs tend to demonstrate acceptable performance in knowledge-based tasks, but significantly worse performance with complex or unstructured clinical tasks, which aligns with our results of decreasing performance as medication complexity increased.

This study has several limitations. The primary purpose of this study was to evaluate baseline performance or internal knowledge of LLMs for identifying DDIs, an important benchmarking task for the safe application of LLMs for medication management. However, it is known that prompt engineering and other LLM methods (e.g., knowledge graphs, multi-agent teams) can improve performance. Second, while these datasets have an advantage in being clinician-developed to aid in clinical relevance and quality, they are relatively small by LLM standards. Finally, DDIs were clinically validated and correlated with drug interaction severity using a standard drug reference; however, additional references for DDIs may have slightly different categorizations for severity and existence of DDIs as new literature is available.

Ultimately, DDI identification was poor amongst all models. Performance decreased as complexity increased, with identification of a DDI in cases of 4–6 medications only 68–80% of the time. This included not identifying category X DDIs (avoid combination) in 24–30% of cases, where potential for patient harm is significant. Considering the average number of medications for an outpatient and inpatient regimen is 4–7 and 7–15, respectively, and the known positive association of adverse drug events with an increasing number of medications, there is concern for using LLMs to identify DDIs, due to the worsened performance with increasing complexity of medication regimen.^34–37^ Unidentified DDIs included interactions such as enoxaparin and alteplase (increased bleeding risk), sulfamethoxazole-trimethoprim and spironolactone (hyperkalemia), dronedarone and azithromycin (QTc prolongation), and phenytoin and apixaban (decreased effectiveness of apixaban).These few examples of undetected DDI pose high risk situations for critical adverse drug events, and would have likely resulted in a clinical pharmacist intervention or discussion with the medical team prior to the initiation of therapy. Poor precision risks identification of a DDI that does not exist. In clinical practice, this could lead to alterations in treatment regimens resulting in reduce clinical outcomes and unnecessary monitoring efforts. Again, it is important to realize that rules-based computer software has 100% performance in this domain, indicating that while LLMs may be a useful tool, clinician back-up using present day standard of care is warranted. Investigating augmented performance of LLMs was beyond the scope of this evaluation, however, future direction of evaluations should involve pretraining with advanced LLM techniques.

## Conclusion

The LLMs had moderate performance on 3 different DDI tasks, with performance generally decreasing with increased complexity (both increasing number of medications and requiring the LLM to specify the interaction severity). Further model improvement is necessary for routine use for identification of clinically relevant DDIs.

## Supplementary Material

Supplement 1

## Figures and Tables

**Table 1.** Description of datasets

	Drug Pair Experiment (n=250)	3 Drug Combination Experiment (n=250)	4–6 Drug Combination Experiment (n=250)
**1 Drug Interaction Present**	125 (50)	250 (100)	250 (100)
**Category A**	125 (50)	0 (0)	0 (0)
**Category B**	0 (0)	0 (0)	0 (0)
**Category C**	40 (16)	82 (32.8)	81 (32.4)
**Category D**	45 (18)	78 (31.2)	77 (30.8)
**Category X**	40 (16)	90 (36)	92 (36.8)
**Number of Medications Per Prompt**	2 (2–2)	3 (3–3)	4 (4–5)

Each experiment contained a total of 250 groups with unique medication scenarios. Experiment “drug-pair” is the only assessment containing 50% of groups with “negative” or “no known interactions”. The remainder of experiments contained drug-drug interaction pairs within C, D, or X interaction categories.

All results as n (%) or median (IQR)

Drug interaction categories are defined as follows:

Category X — Avoid combination: This drug pair should generally not be used together because the clinical risk outweighs any potential benefit.

Category D — Consider therapy modification: The interaction is clinically relevant, and modification of therapy (dose adjustment, substitution, or precautions) should be considered.

Category C — Monitor therapy: An interaction is present. The combination may be used but requires monitoring by a healthcare professional.

Category B — No action needed: An interaction may exist, but no clinical intervention is required.

Category A — No known interaction: There is no known documented interaction between the two drugs.

**Table 2.** Input and output prompts for three drug-drug interaction benchmark experiments

Benchmark	Input	Output
**DDI Pair**	You are a clinical pharmacist specializing in drug-drug interactions. Drug-drug interactions may occur when one medication alters the clinical effect of another (pharmacodynamic interaction) or affects its absorption, distribution, metabolism, or excretion (pharmacokinetic interaction). Drug interaction categories are defined as follows: Category X — Avoid combination: This drug pair should generally not be used together because the clinical risk outweighs any potential benefit. Category D — Consider therapy modification: The interaction is clinically relevant, and modification of therapy (dose adjustment, substitution, or precautions) should be considered. Category C — Monitor therapy: An interaction is present. The combination may be used but requires monitoring by a healthcare professional. Category B — No action needed: An interaction may exist, but no clinical intervention is required. Category A — No known interaction: There is no known documented interaction between the two drugs. Your job is to evaluate whether the drug pair has been appropriately categorized into the correct drug-drug interaction category according to these definitions. Drug Pair: {drug_pair} Proposed Classification: {proposed_category}: {proposed_action} Question: Is this classification correct? A: “CORRECT” B: “INCORRECT” Just return “A” or “B”, with no text around it.	A or B
**DDI 3 Drug Combination**	You are evaluating drug-drug interactions. For the given drug, which drug (A or B) results in a category C, D, or X interaction? Drug interaction categories are defined as follows: Category X — Avoid combination: This drug pair should generally not be used together because the clinical risk outweighs any potential benefit. Category D — Consider therapy modification: The interaction is clinically relevant, and modification of therapy (dose adjustment, substitution, or precautions) should be considered. Category C — Monitor therapy: An interaction is present. The combination may be used but requires monitoring by a healthcare professional. Drug Interaction Category Definition in Use: Category {category_label} Question: Which drug causes a Category {category_label} interaction with {target_drug}? A: candidate_drug_a B: candidate_drug_b Just return “A” or “B” with no text around it.	A or B
**DDI 4–6 Drug Combination**	You are a clinical pharmacist specializing in drug-drug interactions. You will be given a list of medications taken together. Identify two medications that have a category C, D, or X drug interaction. Only identify one drug pair in JSON format. Do not estimate confidence or include any explanation. Drug interaction categories are defined as follows: Category X — Avoid combination: This drug pair should generally not be used together because the clinical risk outweighs any potential benefit. Category D — Consider therapy modification: The interaction is clinically relevant, and modification of therapy (dose adjustment, substitution, or precautions) should be considered. Category C — Monitor therapy: An interaction is present. The combination may be used but requires monitoring by a healthcare professional. Medication List: {medication_lis Format: { “interactions”: [ [“<drug_x>”, “<drug_y>”] ] } Do not include any text before or after the JSON. Do not wrap the JSON in markdown code blocks. Return only the raw JSON object.	Drug X Drug Y

DDI: drug-drug interaction

Table 1 includes each of the prompts utilized in the three drug-drug interaction experiments. Experiment “drug pair” focuses on identifying the presence and appropriate categorization of the drug-drug interaction between 2 medications. The experiment “3 drug combination” prompt queries appropriate identification of a single drug-drug interaction between 3 drugs and classification into the correct category. The experiment “4–6 drug combination” prompt mimics the previous experiment, with a higher complexity, by assessing four to six medications.

**Table 3.** Results

	GPT-4o-mini (n=250)	MedGemma-27B (n=250)	LLaMA3-70B (n=250)
***Drug Pair Experiment (Pointwise)***			
F1	54.5% [53.5, 55.7]	42.2% [37.8, 46.7]	59.7% [56.0, 63.4]
Precision	65.7% [62.8, 68.8]	51.8% [48.9, 54.4]	60.8% [57.8, 63.9]
Recall	46.7% [46.0, 47.5]	38.2% [29.8, 46.6]	61.7% [51.7, 71.7]
Accuracy	61.0% [59.4, 62.6]	50.1% [48.1, 52.0]	59.8% [59.2, 60.5]
Self-Consistency Score	208/500 (41.6)	155/500 (31.0)	215/500 (43.0)
***3 Drug Combination Experiment (Pairwise)***			
Accuracy	86.6% [82.8, 91.2]	84.9% [78.4, 87.6]	81.5% [93.2, 98.4]
Self-Consistency Score	217 (86.6)	212 (84.9)	204 (81.5)
Category C (n=82)			
Accuracy	73.3% [63.4, 81.7]	76.7% [59.8, 79.3]	76.8% [86.6, 97.6]
Self-Consistency Score	60 (73.3)	63 (76.8)	63 (76.8)
Category D (n=78)			
Accuracy	92.3% [85.9, 97.4]	89.2% [82.1, 96.2]	89.0% [93.6, 100.0]
Self-Consistency Score	72 (92.3)	70 (89.7)	69 (88.5)
Category X (n=90)			
Accuracy	93.8% [91.1, 98.9]	88.6% [82.2, 95.6]	79.3% [94.4, 100.0]
Self-Consistency Score	84 (93.3)	80 (88.9)	71 (78.9)
***4–6 Drug Combination Experiment (Listwise)***			
Accuracy	71.3% [66.4, 77.6]	80.0% [75.2, 85.2]	68.6% [63.2, 74.4]
Self-Consistency Score	178 (71.2)	200 (80.0)	172 (68.8)
Category C (n=82)			
Accuracy	65.0% [56.8, 76.5]	73.8% [65.4, 84.0]	58.3% [48.1, 67.9]
Self-Consistency Score	53 (65.4)	60 (74.1)	47 (58.0)
Category D (n=78)			
Accuracy	72.0% [62.0, 80.5]	85.0% [77.2, 91.3]	73.0% [63.0, 81.6]
Self-Consistency Score	66 (71.7)	78 (84.8)	67 (72.8)
Category X (n=90)			
Accuracy	77.2% [67.5, 87.0]	80.5% [72.7, 89.6]	74.3% [63.6, 84.4]
Self-Consistency Score	59 (76.6)	62 (80.5)	57 (74.0)

All data reported as mean % [95% confidence interval] or n (%)

Self-consistency = (number of instances with all 9 runs correct) / (total number of instances)

Recall: true positive/ (true positive + false negative). Low recall indicates many false negatives.

Precision: true positive/ (true positive + false positive). Low precision indicates many false positives

F1: 2 × (precision × recall)/(precision + recall). Low F1 indicates many false negatives, false positives, or both.

Accuracy = (True Positive + True Negative) / (True Positive + True Negative + False Positive + False Negative), this shows the fraction of correct predictions

Drug interaction categories are defined as follows:

Category X — Avoid combination: This drug pair should generally not be used together because the clinical risk outweighs any potential benefit.

Category D — Consider therapy modification: The interaction is clinically relevant, and modification of therapy (dose adjustment, substitution, or precautions) should be considered.

Category C — Monitor therapy: An interaction is present. The combination may be used but requires monitoring by a healthcare professional.
