# Supplementary material for: Drug-drug interaction identification using large language models
Source: medRxiv. 2025 Dec 29:2025.12.03.25341549. Preprint. [Version 2] doi: 10.64898/2025.12.03.25341549 (PMC12772686; doi:10.64898/2025.12.03.25341549)
Supplement: Supplement 1 [file media-1.docx]

# Supplementary Appendix

Table of Contents

[Supplementary Appendix 1](#_Toc216243790)

[TRIPOD+LLM Checklists 2](#_Toc216243791)

[PER-RUN METRICS 8](#_Toc216243792)

## TRIPOD+LLM Checklists


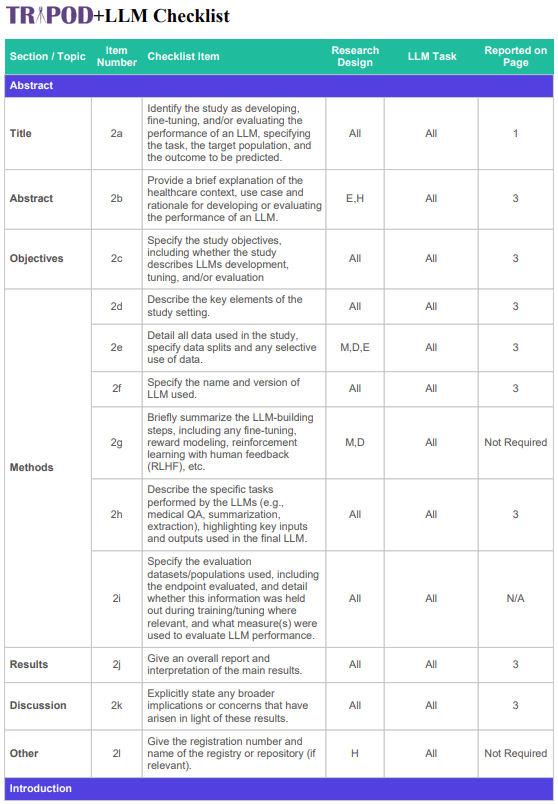


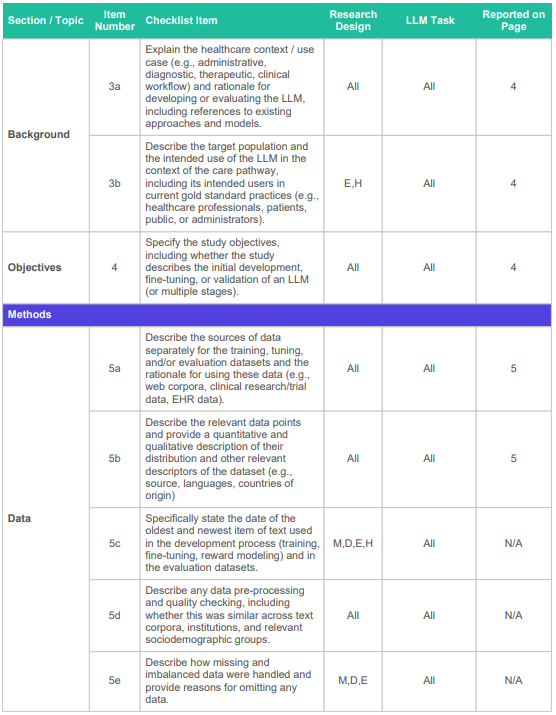


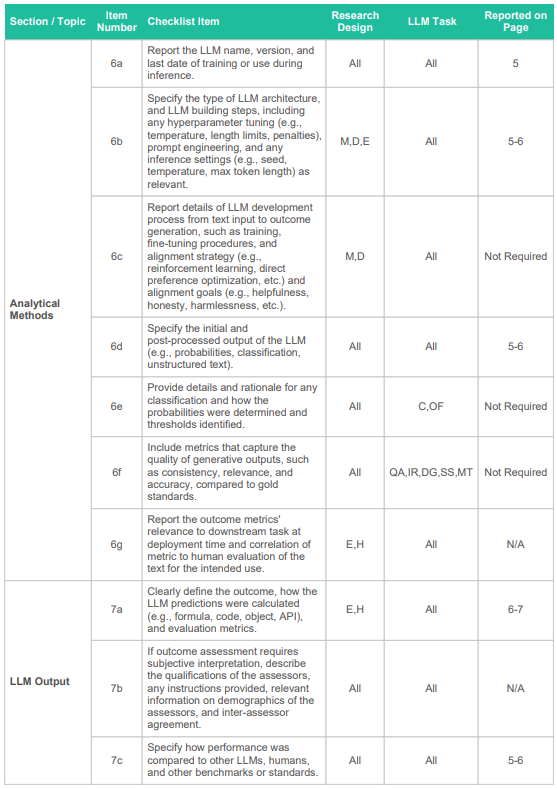


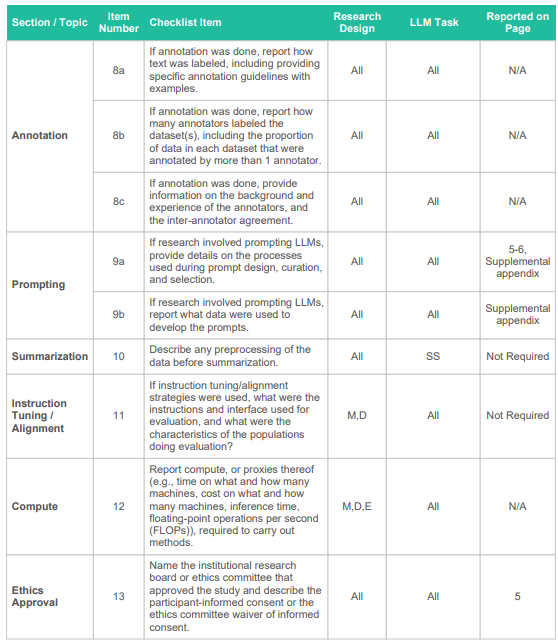


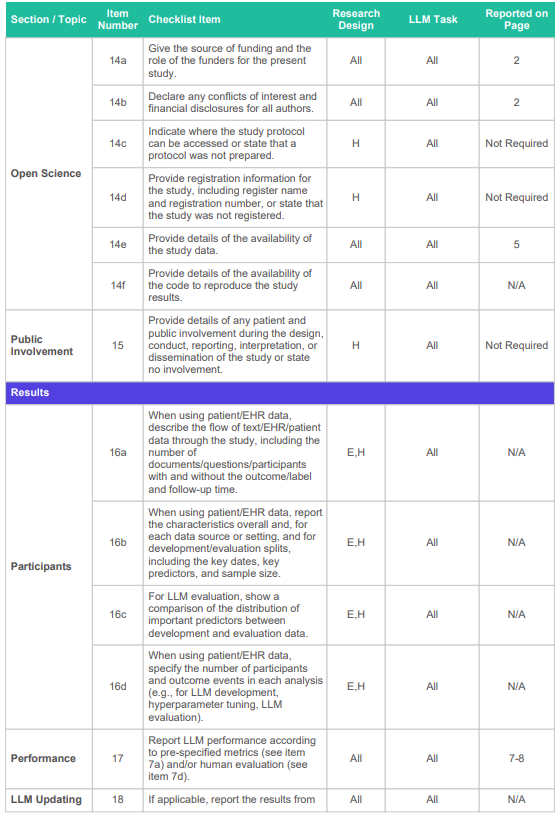


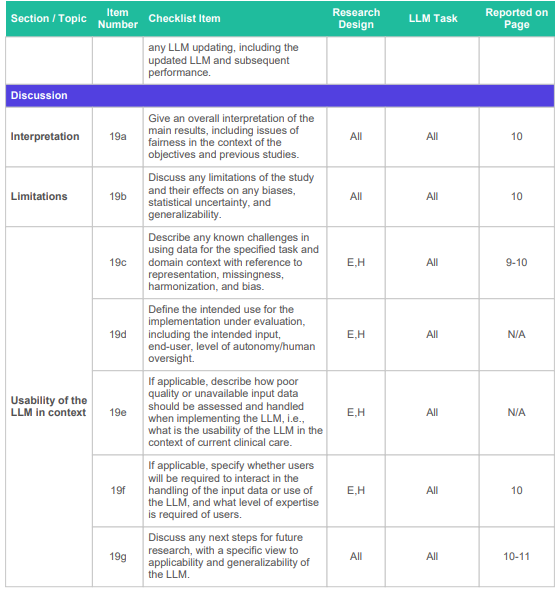


## PER-RUN METRICS

CONSISTENCY RESULTS - PER-RUN METRICS
 ================================================================================

This file contains detailed metrics for each individual run.
 Main screen shows only averaged results with Self-Consistency.


POINTWISE

Run: ptrue_pair_gpt4o_shuffle_false_run1
 Model TP FP TN FN Precision Recall F1 Accuracy
 gpt-4o-mini 117 46 204 133 0.718 0.468 0.567 0.642
 LLaMA3-70B 113 58 192 137 0.661 0.452 0.537 0.610
 MedGemma-27B 128 148 102 122 0.464 0.512 0.487 0.460

Run: ptrue_pair_gpt4o_shuffle_true_run1
 Model TP FP TN FN Precision Recall F1 Accuracy
 gpt-4o-mini 116 76 174 134 0.604 0.464 0.525 0.580
 LLaMA3-70B 187 142 108 63 0.568 0.748 0.646 0.590
 MedGemma-27B 68 54 196 182 0.557 0.272 0.366 0.528

Run: ptrue_pair_gpt4o_shuffle_false_run2
 Model TP FP TN FN Precision Recall F1 Accuracy
 gpt-4o-mini 120 49 201 130 0.710 0.480 0.573 0.642
 LLaMA3-70B 113 58 192 137 0.661 0.452 0.537 0.610
 MedGemma-27B 133 144 106 117 0.480 0.532 0.505 0.478

Run: ptrue_pair_gpt4o_shuffle_true_run2
 Model TP FP TN FN Precision Recall F1 Accuracy
 gpt-4o-mini 121 73 177 129 0.624 0.484 0.545 0.596
 LLaMA3-70B 188 143 107 62 0.568 0.752 0.647 0.590
 MedGemma-27B 68 57 193 182 0.544 0.272 0.363 0.522

Run: ptrue_pair_gpt4o_shuffle_false_run3
 Model TP FP TN FN Precision Recall F1 Accuracy
 gpt-4o-mini 115 50 200 135 0.697 0.460 0.554 0.630
 LLaMA3-70B 113 58 192 137 0.661 0.452 0.537 0.610
 MedGemma-27B 128 148 102 122 0.464 0.512 0.487 0.460

Run: ptrue_pair_gpt4o_shuffle_true_run3
 Model TP FP TN FN Precision Recall F1 Accuracy
 gpt-4o-mini 120 75 175 130 0.615 0.480 0.539 0.590
 LLaMA3-70B 188 143 107 62 0.568 0.752 0.647 0.590
 MedGemma-27B 67 52 198 183 0.563 0.268 0.363 0.530

Run: ptrue_pair_gpt4o_shuffle_false_run4
 Model TP FP TN FN Precision Recall F1 Accuracy
 gpt-4o-mini 113 48 202 137 0.702 0.452 0.550 0.630
 LLaMA3-70B 112 59 191 138 0.655 0.448 0.532 0.606
 MedGemma-27B 132 141 109 118 0.484 0.528 0.505 0.482

Run: ptrue_pair_gpt4o_shuffle_true_run4
 Model TP FP TN FN Precision Recall F1 Accuracy
 gpt-4o-mini 117 71 179 133 0.622 0.468 0.534 0.592
 LLaMA3-70B 188 145 105 62 0.565 0.752 0.645 0.586
 MedGemma-27B 69 54 196 181 0.561 0.276 0.370 0.530

Run: ptrue_pair_gpt4o_shuffle_true_run5
 Model TP FP TN FN Precision Recall F1 Accuracy
 gpt-4o-mini 112 68 182 138 0.622 0.448 0.521 0.588
 LLaMA3-70B 187 142 108 63 0.568 0.748 0.646 0.590
 MedGemma-27B 66 56 194 184 0.541 0.264 0.355 0.520

--------------------------------------------------------------------------------


PAIRWISE

Run: discrimination_gpt4o_shuffle_false_run1
 Model TP FP TN FN Precision Recall F1 Accuracy
 gpt-4o-mini 224 0 0 26 1.000 0.896 0.945 0.896
 LLaMA3-70B 156 0 0 94 1.000 0.624 0.768 0.624
 MedGemma-27B 228 0 0 22 1.000 0.912 0.954 0.912

Run: discrimination_gpt4o_shuffle_true_run1
 Model TP FP TN FN Precision Recall F1 Accuracy
 gpt-4o-mini 212 0 0 38 1.000 0.848 0.918 0.848
 LLaMA3-70B 241 0 0 9 1.000 0.964 0.982 0.964
 MedGemma-27B 199 0 0 51 1.000 0.796 0.886 0.796

Run: discrimination_gpt4o_shuffle_false_run2
 Model TP FP TN FN Precision Recall F1 Accuracy
 gpt-4o-mini 224 0 0 26 1.000 0.896 0.945 0.896
 LLaMA3-70B 158 0 0 92 1.000 0.632 0.775 0.632
 MedGemma-27B 228 0 0 22 1.000 0.912 0.954 0.912

Run: discrimination_gpt4o_shuffle_true_run2
 Model TP FP TN FN Precision Recall F1 Accuracy
 gpt-4o-mini 211 0 0 39 1.000 0.844 0.915 0.844
 LLaMA3-70B 241 0 0 9 1.000 0.964 0.982 0.964
 MedGemma-27B 199 0 0 51 1.000 0.796 0.886 0.796

Run: discrimination_gpt4o_shuffle_false_run3
 Model TP FP TN FN Precision Recall F1 Accuracy
 gpt-4o-mini 225 0 0 25 1.000 0.900 0.947 0.900
 LLaMA3-70B 157 0 0 93 1.000 0.628 0.771 0.628
 MedGemma-27B 230 0 0 20 1.000 0.920 0.958 0.920

Run: discrimination_gpt4o_shuffle_true_run3
 Model TP FP TN FN Precision Recall F1 Accuracy
 gpt-4o-mini 211 0 0 39 1.000 0.844 0.915 0.844
 LLaMA3-70B 241 0 0 9 1.000 0.964 0.982 0.964
 MedGemma-27B 203 0 0 47 1.000 0.812 0.896 0.812

Run: discrimination_gpt4o_shuffle_false_run4
 Model TP FP TN FN Precision Recall F1 Accuracy
 gpt-4o-mini 225 0 0 25 1.000 0.900 0.947 0.900
 LLaMA3-70B 158 0 0 92 1.000 0.632 0.775 0.632
 MedGemma-27B 226 0 0 24 1.000 0.904 0.950 0.904

Run: discrimination_gpt4o_shuffle_true_run4
 Model TP FP TN FN Precision Recall F1 Accuracy
 gpt-4o-mini 207 0 0 43 1.000 0.828 0.906 0.828
 LLaMA3-70B 241 0 0 9 1.000 0.964 0.982 0.964
 MedGemma-27B 199 0 0 51 1.000 0.796 0.886 0.796

Run: discrimination_gpt4o_shuffle_true_run5
 Model TP FP TN FN Precision Recall F1 Accuracy
 gpt-4o-mini 210 0 0 40 1.000 0.840 0.913 0.840
 LLaMA3-70B 241 0 0 9 1.000 0.964 0.982 0.964
 MedGemma-27B 198 0 0 52 1.000 0.792 0.884 0.792

--------------------------------------------------------------------------------


LISTWISE

Run: verbalize_gpt4o_shuffle_false_run1
 Model TP FP TN FN Precision Recall F1 Accuracy
 gpt-4o-mini 175 0 0 75 1.000 0.700 0.824 0.700
 LLaMA3-70B 171 0 0 79 1.000 0.684 0.812 0.684
 MedGemma-27B 201 0 0 49 1.000 0.804 0.891 0.804

Run: verbalize_gpt4o_shuffle_true_run1
 Model TP FP TN FN Precision Recall F1 Accuracy
 gpt-4o-mini 177 0 0 73 1.000 0.708 0.829 0.708
 LLaMA3-70B 171 0 0 79 1.000 0.684 0.812 0.684
 MedGemma-27B 200 0 0 50 1.000 0.800 0.889 0.800

Run: verbalize_gpt4o_shuffle_false_run2
 Model TP FP TN FN Precision Recall F1 Accuracy
 gpt-4o-mini 178 0 0 72 1.000 0.712 0.832 0.712
 LLaMA3-70B 171 0 0 79 1.000 0.684 0.812 0.684
 MedGemma-27B 199 0 0 51 1.000 0.796 0.886 0.796

Run: verbalize_gpt4o_shuffle_true_run2
 Model TP FP TN FN Precision Recall F1 Accuracy
 gpt-4o-mini 176 0 0 74 1.000 0.704 0.826 0.704
 LLaMA3-70B 173 0 0 77 1.000 0.692 0.818 0.692
 MedGemma-27B 201 0 0 49 1.000 0.804 0.891 0.804

Run: verbalize_gpt4o_shuffle_false_run3
 Model TP FP TN FN Precision Recall F1 Accuracy
 gpt-4o-mini 177 0 0 73 1.000 0.708 0.829 0.708
 LLaMA3-70B 174 0 0 76 1.000 0.696 0.821 0.696
 MedGemma-27B 196 0 0 54 1.000 0.784 0.879 0.784

Run: verbalize_gpt4o_shuffle_true_run3
 Model TP FP TN FN Precision Recall F1 Accuracy
 gpt-4o-mini 180 0 0 70 1.000 0.720 0.837 0.720
 LLaMA3-70B 171 0 0 79 1.000 0.684 0.812 0.684
 MedGemma-27B 201 0 0 49 1.000 0.804 0.891 0.804

Run: verbalize_gpt4o_shuffle_false_run4
 Model TP FP TN FN Precision Recall F1 Accuracy
 gpt-4o-mini 180 0 0 70 1.000 0.720 0.837 0.720
 LLaMA3-70B 171 0 0 79 1.000 0.684 0.812 0.684
 MedGemma-27B 202 0 0 48 1.000 0.808 0.894 0.808

Run: verbalize_gpt4o_shuffle_true_run4
 Model TP FP TN FN Precision Recall F1 Accuracy
 gpt-4o-mini 180 0 0 70 1.000 0.720 0.837 0.720
 LLaMA3-70B 171 0 0 79 1.000 0.684 0.812 0.684
 MedGemma-27B 201 0 0 49 1.000 0.804 0.891 0.804

Run: verbalize_gpt4o_shuffle_true_run5
 Model TP FP TN FN Precision Recall F1 Accuracy
 gpt-4o-mini 182 0 0 68 1.000 0.728 0.843 0.728
 LLaMA3-70B 171 0 0 79 1.000 0.684 0.812 0.684
 MedGemma-27B 199 0 0 51 1.000 0.796 0.886 0.796

--------------------------------------------------------------------------------
